# Seasonal variations of vitamin D and its relation to lipid profile in Iranian children and adults

**DOI:** 10.1186/s41043-017-0096-y

**Published:** 2017-05-22

**Authors:** Sakineh Nouri Saeidlou, Davoud Vahabzadeh, Fariba Babaei, Zakaria Vahabzadeh

**Affiliations:** 1 Food and Beverages Safety Research Center, Urmia University of Medical Sciences, Urmia, Iran; 2 Maternal and Child Obesity Research Center (MCORC), Urmia University of Medical Sciences, Urmia, Iran; 3 Deputy of Health, Urmia University of Medical Sciences, Urmia, Iran; 4 Liver & Digestive Research Center, Kurdistan University of Medical Sciences, Sanandaj, Iran

**Keywords:** Vitamin D, Lipid profile, Child, Adult, Iran

## Abstract

**Background:**

Vitamin D has a multitude of functional properties and acts like a hormone in the body. Its effect on the lipid profile is one of the proposed mechanisms for its relationship with many disorders during its deficiency. But, this relationship is still conflicting and debatable, so this study was conducted to determine the association between serum level of vitamin D and lipid profiles, including serum concentrations of cholesterol, triglyceride (TG), HDL, and LDL in healthy subjects.

**Methods:**

In this cross-sectional study, 541 volunteers with age of 5–60 years from normal and healthy subjects were selected via random sampling. Demographics and history of daily or weekly sunlight exposures were recorded. Measuring vitamin D was done in two consecutive seasons: winter and summer. Ten milliliters of peripheral venous blood sample was withdrawn after an overnight fasting. Serum levels of 25(OH) D (25, hydroxy vitamin D3) were measured using the enzyme-linked immunosorbent assay (ELISA), and the Confirmatory test was done by high-performance liquid chromatography (HPLC).

**Results:**

Mean age in the total mixed population was 30.83 ± 14.02 years. Subjects were 50.5% male and 49.5% female. Mean 25(OH) D in the total population for winter and summer were 45.8 ± 24.26 ng/ml and 55.24 ± 37.47 ng/ml respectively. In the total population, 38.08% were vitamin D deficient. Comparing serum lipid levels in the summer and winter showed a significant difference for cholesterol, LDL, and HDL, but no significant effect was found for TG. Analysis for the comparison of lipid profiles between the two genders in winter showed that there were significant differences in all lipid profiles except for LDL, while such analysis for summer revealed significant difference just for TG. In multivariate analysis, there was a significant mean difference only for LDL in subgroups with vitamin D insufficiency and deficiency. There was no correlation between Vitamin D and lipid profiles.

**Conclusions:**

Vitamin D is different between the two seasons regardless of gender variations. Its status showed some significant relationship with some lipid profiles (cholesterol, LDL, and HDL) during the two seasons. There were different results among winter and summer based on the gender.

## Background

Vitamin D acts in the body like a hormone with a multitude of functions. The main source of vitamin D is solar UV radiation, which converts 7-dehydrocholesterol to pro-vitamin D [1]. Dehydrocholesterol is in turn modified to vitamin D (cholecalciferol) given normal skin temperature. There are few natural dietary sources of vitamin D, which can be found in fatty fish like salmon and herring and in cod liver oil [2].

Serum 25(OH) D (25, hydroxy vitamin D3), the recognized biomarker of vitamin D status best reflects the vitamin D status of the body [3]. Vitamin D deficiency can be defined as vitamin D serum levels less than 27.5 ng/ml. Vitamin D deficiency is a common disorder, found in all age groups and in both genders [4]. Several mechanisms may be related to 25(OH) D deficiency and variation in lipid profile, but there is no consensus on the biological plausibility [5, 6].

Worldwide, the prevalence of vitamin D deficiency is 50% in the elderly [7], affecting 30 to 50% of the adult US population [8] and 20–30% of the adult European population [9], with increased occurrence in high- and low-latitude countries. In one multi-center osteoporosis study from Iran, the prevalence of mild, moderate, and severe vitamin D deficiency has been estimated at respectively 47.2, 45.7, and 44.2% among women and 54.2, 41.2, and 37.5% among men in a ≤60-year-old population [5, 10].

From results of cross-sectional data used for meta-analysis where the majority of the study results indicated that serum 25(OH) D is directly associated with serum HDL-C and inversely related to TC, LDL, and triglyceride (TG), it is important to highlight that higher serum 25(OH) D is related to a more favorable lipid profile in the pediatric age groups [10].

The relationship between vitamin D status and lipid profile is unclear. Some epidemiological studies suggest an inverse association between circulating levels of 25(OH) D and cardiovascular risk biomarkers [11, 12], but other studies have not supported the benefit of 25(OH) D supplementation to improve the blood lipid profile [13, 14].

Vitamin D can be affected by environmental factors, such as exposure to ultraviolet light and consumption of foods rich in fat-soluble vitamin D staples (e.g., oily sea fish, meat, and eggs), so repletion of this vitamin’s reserves can be done by such factors [12].

Hypovitaminosis D was shown to be associated not only with lowered insulin secretion and sensitivity but also with adverse effects on TG, total cholesterol, and LDL-cholesterol and HDL-cholesterol concentrations in a study of healthy men and women from several racial and ethnic groups [15].

It has been proposed that vitamin D has a link with different cardiovascular diseases such as HTN, CVD through its different role in endothelial function, blood pressure control, calcification of the coronary vessels, and increased vascular resistance [5, 8, 16]. Also, the effect of vitamin D on the regulation of the lipid profile is one of the proposed mechanisms for the relationship between vitamin D deficiency and the abovementioned disorders. The aim of our study is to determine the association between the serum level of vitamin D and lipid profiles, including serum concentrations of cholesterol, TG, HDL, and LDL in the north of Iran in healthy subjects.

## Methods

This cross-sectional study was performed in West Azerbaijan province, a region of the Mediterranean with spring rain at latitude 37°, as a part of a national survey on food and nutrition in the northwest of Iran during winter and summer, 2015. Five hundred and forty-one volunteers with age of 5–60 years from normal and healthy subjects were entered. After dividing the urban area into five different sectors based on socioeconomic status, one health center from each sector was selected randomly. In proportion to the covered child and adult’s population in each center, subjects who were eligible for the study were included consequently, after obtaining written consent.

Exclusion criteria were pregnancy, lactation, use of drugs affecting the lipid profile or calcium and bone metabolism, chronic disorders of the liver or kidney, endocrinology disorders such as hypo- or hyperthyroidism and hyperparathyroidism, smoking, insulin injection, use of anticonvulsive drugs, and vitamin D or calcium supplementation.

Demographics and history of daily or weekly sunlight exposures were recorded in the pre-prepared questionnaire. After overnight fasting, 10 ml of peripheral venous blood sample was withdrawn. Blood samples were centrifuged at 3000 rpm for 10 min and stored at −20 °C. Serum levels of 25(OH) D were measured using the enzyme-linked immunosorbent assay (ELISA), and the confirmatory test was done by high-performance liquid chromatography (HPLC) which measured 25(OH) D. Measurements were done in two consecutive seasons: winter and summer (2014) for the same subjects.

The normal range for 25(OH) D was considered as 50–75 ng/ml. A 25(OH) D level of lower than 27.5 ng/ml was considered as vitamin D deficiency, levels between 27.5 and 49.99 ng/ml considered as insufficient, and levels of equal and more than 50 ng/ml as sufficient. Plasma total cholesterol, HDL-C, and triglyceride concentrations were measured in duplicate using enzymatic kits, standardized reagents, and standards (Pars Azmoon Co., Tehran, Iran) and autoanalyzer (Selectra E, Vitalab, Holliston, Netherlands). LDL-C concentration was calculated using the Friedewald equation [13, 17].

All continuous values are expressed as mean ± SD, and categorical variables were presented as a percentage. The independent *t* test was employed to compare differences between the means of continuous variables between two sexes and the season groups. Multivariate analysis and correlation were performed with data to extract associations between vitamin D and different above lipid profiles. One-way ANOVA was used for comparing serum vitamin D and lipid fraction means between three subcategories. Post hoc Tukey test was used for pairwise comparison after ANOVA test. Correlation analysis was recruited for detecting correlations between vitamin D and lipid profiles in the total population with or without considering the season. *P* values less than 0.05 were considered statistically significant. Data were analyzed by SPSS statistical software (version 21.0, SPSS Inc.).

## Results and discussion

This cross-sectional study was conducted in the northwest of Iran in population groups with different age ranges from 5 to 60 years old. We studied this target population while considering two age subcategories: ≤18-year-old and >18-year-old study populations with two different seasonal categories. Also, another analysis was performed based on sex subgroup classification for different age groups. Mean age in total mixed population was 30.83 ± 14.02 years. In the ≤18 years population, mean age was 10.94 ± 3.21 years, and for the >18 years population, it was 37.89 ± 8.86 years. In the less than 18-year-old category, 55.2% were males and 44.8% female. In the above-18-year-old category, percentages were 45.8 and 54.2% respectively. Mean 25(OH) D in the total population for winter and summer were 45.8 ± 24.26 ng/ml and 55.24 ± 37.47 ng/ml respectively.

The prevalence of vitamin D deficiency in males and females was 6.9 and 27.9%, respectively, and the insufficiency of vitamin D was 28.2 and 35.7% respectively. Also, the prevalence of vitamin D deficiency in the total studied population was 38.08% and the overall vitamin D deficiency and insufficiency according to seasonal categorization were 87.41 and 49.82% in winter and summer, respectively (Table 1).

**Table 1:** Vitamin D status in two different seasonal categories in summer and winter

Vitamin D status	Summer *N* (%)	Winter *N* (%)	Total *N* (%)
Deficiency	48 (17.70)	158 (58.52)	206 (38.08)
Insufficiency	87 (32.12)	78 (28.89)	165 (30.50)
Sufficiency	136 (50.18)	34 (12.59)	170 (31.42)
Total	271 (100)	270 (%100)	541 (%100)

**Fig. 1:**
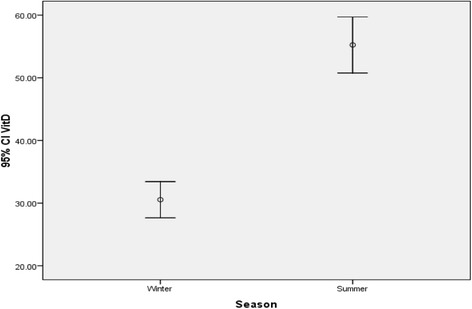
Comparing mean differences between winter and summer for 25(OH) D (*P* < 0.001)

**Fig. 2:**
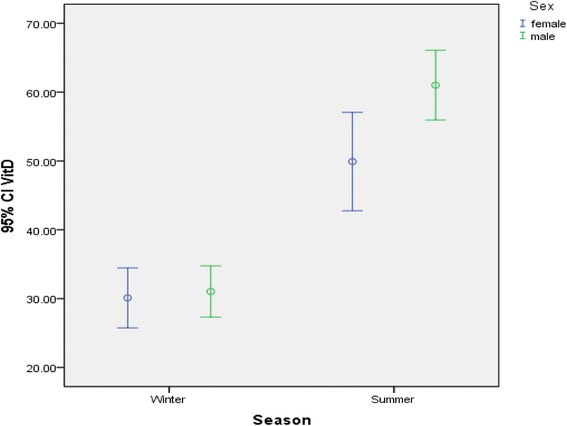
Comparing mean differences between winter and summer for 25(OH) D in different sexes (*P* < 0.001)

In the total population, mean differences between winter and summer for 25(OH) D were significant (*P* < 0.001). Mean 25(OH) D for the population in winter and summer was 30.96 ± 24.26 ng/ml and 55.81 ± 37.39 ng/ml respectively (Fig. 1). Also, additional analysis for comparing mean differences between winter and summer for males and females was significant (*P* < 0.001) (Fig. 2).

The current study revealed that differences of average serum levels of lipids in the studied populations in summer and winter were significant for cholesterol, LDL, and HDL, but no significant difference was found for TG. Analysis for comparing lipid profiles between two genders in winter showed that there were significant differences in all lipid profiles except for LDL, while such analysis for summer revealed that there were no significant differences in lipid profiles except for TG (Table 2).

**Table 2:** Comparing mean lipid profiles between two genders within different seasons

Variable	Sex	Mean ± SD (winter)	*P* value	Mean ± SD (summer)	*P* value
TG	Female	108.29 ± 55.08	0.007	116.94 ± 66.46	0.05
	Male	158.04 ± 23.08		162.24 ± 258.58	
CHOL	Female	159.18 ± 35.27	0.03	172.66 ± 32.19	0.60
	Male	167.93 ± 44.41		175.11 ± 45.68	
LDL	Female	91.07 ± 29.02	0.4	101.53 ± 25.78	0.46
	Male	93.70 ± 30.06		99.03 ± 29.81	
HDL	Female	46.42 ± 10.97	0.016	47.73 ± 11.29	0.08
	Male	43.17 ± 14.19		44.86 ± 15.56	

According to the analysis of variances, mean differences for lipid profiles between three groups in different levels of vitamin D indicated that in subgroups with insufficient and deficient levels of vitamin D, there was a significant mean difference only for LDL. Post hoc Tukey test for comparing mean differences are shown in Table 3.

**Table 3:** Post hoc Tukey test results for comparing mean LDL between two different groups based vitamin D status

	Mean differences	Sig	CI
Deficiency/insufficiency	7.76	0.02	0.93–14.6
Deficiency/adequacy	6.00	0.09	7.8–12.78
Insufficiency/deficiency	1.76	0.83	5.41–8.94

**Fig. 3:**
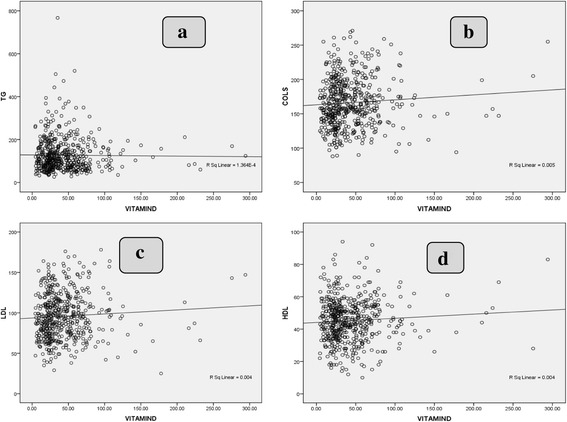
**a** Correlation between 25(OH) D level and triglyceride level (*r* = −0.029; *P* = 0.638). **b** Correlation between 25(OH) D level and cholesterol level (*r* = −0.013; *P* = 0.826). **c** Correlation between 25(OH) D level and LDL level (*r* = −0.015; *P* = 0.805) **d** Correlation between 25(OH) D level and HDL level (*r* = 0.043; *P* < 0.479)

Correlation analysis among study populations is shown in Fig. 3, with no correlation between 25(OH) D and TG, LDL-C, cholesterol, and HDL.

Serum 25(OH) D is a well-recognized biomarker of vitamin D status that best reflects the vitamin D content of the body [3]. Vitamin D can be affected by environmental factors, such as exposure to ultraviolet light and consumption of foods rich in fat-soluble vitamin D staples (e.g., oily sea fish, meat, and eggs), so repletion of this vitamin’s reserves can be done by such factors [12].

This study has established that vitamin D deficiency is more prevalent in the over-18-year-old subgroup but more so in women than in men. Also, vitamin D deficiency was higher in winter than summer. Different studies have reported different values for vitamin D deficiency prevalence. The discrepancies of studies might be partly explained by confounders as age and gender. Similar studies have reported such inequality distribution of vitamin D deficiency and insufficiency among two different sexes in Asian Indians [18]. However, some reports have established equal distribution between different sexes in Pakistan [19].

Our finding confirmed an inequality of vitamin D deficiency in subjects between two different genders and seasons. This study like other studies [16] reported that seasonal variation in vitamin D levels can be cited as reasons for overestimation of vitamin D deficiency [20–22], so vitamin D status reporting should be done considering season and gender variations.

Results from studies might be confounded by studied population heterogeneity and the confounders such as parathyroid hormone (PTH), calcium, physical activity, kidney function, sex, age, sun exposure status, and clothing pattern [7, 10]. The most reason for seasonal variation may be due to less exposure to the sun in some regions of the world and reduced intake of vitamin D. Also changes in estrogen level with increasing age in women and consequently changes in Ca and bone metabolism can be related to vitamin D variation between men and women. Another factor affecting vitamin status and its reduction in females can be women garments in Islamic territories [3, 10].

In some studies, the positive effect on lipid profiles has been proposed for vitamin D, but it is not clear whether or not the beneficial effects of vitamin D are due to the hormone itself or its association with calcium metabolism [6, 23]. Calcium acts to form insoluble soaps with dietary fat, preventing its absorption, thus modulating the effect of high dietary fat on blood lipid concentrations [2].

There are different studies with different results and in some instances conflicting results on the relationship between serum levels of vitamin D and lipid profiles. Ford and colleagues, in their NHANES III study, found a negative association between serum levels of 25(OH) D and TG in patients with hypertriglyceridemia, while this relationship was not observed with regard to HDL cholesterol in healthy subjects [24].

In order to determine relationships between 25(OH) D levels and components of the lipid profile status, average serum levels of lipids in the studied populations in summer and winter were significant for cholesterol, LDL, and HDL, but no significant effect was found for TG. Analysis for comparing lipid profiles between two genders in summer showed that there were significant differences in all lipid profiles except for LDL, while such analysis for winter revealed that there were no significant differences in lipid profiles except for TG.

The possible mechanism for the lowering effect of vitamin D can be included as the first mechanism; vitamin D increases serum calcium by enhancing intestinal calcium absorption. This calcium could then reduce serum triglycerides by reducing hepatic triglyceride formation and secretion [25]. The second mechanism is that vitamin D has a suppressive effect on serum PTH concentration. When heparin lipolytic activity decreased by increased PTH concentration, a lowered level of PTH may reduce serum triglycerides by increasing peripheral uptake. In addition to the above mechanisms, other mechanisms have been implicated. Vitamin D may regulate triglycerides metabolism through of increased expression of VLDL cholesterol gene receptors [6]. Another possible mechanism to explain the association between 25(OH) D and triglycerides would be through insulin resistance: when vitamin D deficiency is present, the risk of insulin resistance increases and this is associated with an elevation of levels of VLDL cholesterol and triglycerides [26].

In some cross-sectional studies, an association between an increase in vitamin D level and a decline in LDL, cholesterol, and TG and an increase in HDL have been proposed [5, 6, 13, 14, 27], and so far, intervention trials have not clarified the relationship between serum lipids and vitamin D-calcium supplementation for improving lipid profiles [13, 28].

In the Kelishadi review [10], inverse weak significant association between 25(OH) D and triglycerides, total cholesterol, and LDL-C and direct association with HDL-C was showed, but in the Jorde and Grimnes review [15], five of the seven papers reported positive associations of vitamin D level with LDL but only one being statistically significant, while three reported negative associations, with one being significant.

A meta-analysis by Wang et al. observed a slight effect of vitamin D supplementation on LDL, but no effect on TC, HDL, or TG [11].

Our result-based correlation analysis showed that there was no correlation between vitamin D and any of the lipid profile parameters. In other studies such as Johnson et al. and Rusconi et al. [20, 29], such results have been presented where there were no statistically significant correlations between 25(OH) D levels and total serum cholesterol. But in the Rajakumar et al. study, there were positive correlations between plasma 25(OH) D and HDL cholesterol in black and white children [3].

One of the limitations of this study was the lack of dietary intake and physical activity assessment besides the evaluation of serum levels of vitamin D. Another limitation in this study was its implementation just for an urban area since those who live in rural areas can benefit from higher physical activity and sun exposure.

## Conclusions

This study showed that serum levels of vitamin D can be different in the two seasons regardless of gender variations. Also, its association with a lipid profile is still conflicting and debatable. To find the true relationship between vitamin D status and lipid profile, alongside with full control of confounding factors, group synchronization in randomized controlled trials is recommended.

## Abbreviations

25(OH) D: 25, hydroxy vitamin D3
CVD: Cerebrovascular disease
ELISA: Enzyme-linked immunosorbent assay
HDL: High-density lipoprotein
HPLC: High-performance liquid chromatography
LDL: Low-density lipoprotein
NHANES III: National Health and Nutrition Examination Survey III
PTH: Parathyroid hormone
TG: Triglyceride
